# Defect Dynamics and Solution‐Processed Interconnects in Perovskite‐Organic Tandem Solar Cells

**DOI:** 10.1002/advs.202519528

**Published:** 2025-12-12

**Authors:** Yingjie Hu, Qianyi Li, Kaifeng Jing, Jiangsheng Yu, Fuyi Zhou, Zhenhai Ai, You Chen, Yue Zhao, Yijia Zhang, Zhenyi Ni, Yang Bai, Gang Li, Guang Yang

**Affiliations:** ^1^ Department of Electrical and Electronic Engineering The Hong Kong Polytechnic University Hung Hom, Kowloon Hong Kong 999077 China; ^2^ Photonic Research Institute (PRI) Research Institute of Smart Energy (RISE) Research Institute for Advanced Manufacturing (RIAM) The Hong Kong Polytechnic University Hung Hom, Kowloon Hong Kong 999077 China; ^3^ Faculty of Materials Science and Energy Engineering Shenzhen University of Advanced Technology Shenzhen 518107 China; ^4^ State Key Laboratory of Silicon and Advanced Semiconductor Materials & School of Materials Science and Engineering Zhejiang University Hangzhou 310027 China; ^5^ Institute of Technology for Carbon Neutrality Shenzhen Institutes of Advanced Technology (SIAT) Chinese Academy of Sciences Shenzhen 518055 China

**Keywords:** Defects, interconnecting layers, perovskite‐organic tandem solar cells, stability, voltage loss

## Abstract

Perovskite and organic semiconductors exhibit analogous properties, including bandgap tunability, low‐temperature solution processing, and high potential for lightweight applications. These similarities render them highly attractive for being integrated in multijunction architecture: perovskite‐organic tandem solar cells (POTSCs). Nevertheless, the efficiency of POTSCs is limited by electrical losses, which stem from both the wide‐bandgap (WBG) perovskite layers and the interconnecting layers (ICLs) between two subcells. These two essential components also constrain the tandem device stability. In this study, the underlying cause of open‐circuit voltage (*V*_OC_) losses in WBG perovskites is identified, which is ascribed to the presence of mobile defects distributed at surface regions. An employ effective passivation agent with functional chemical groups is further employed to facilitate the healing of the mobile defects, thereby enhancing the *V*_OC_ to 1.35 V for WBG perovskite solar cells with a bandgap of 1.81 eV. Subsequently, solution‐processed graphene oxide layer ICLs are developed for tandem application, which not only reduces electrical losses but also improves tandem device stability. The synergistic integration of these two strategies has enabled POTSCs to surpass 25% efficiency while simultaneously achieving enhanced operational stability.

## Graphical abstract

In this study, we identify the underlying cause of efficiency losses in WBG perovskites, which is ascribed to the presence of mobile defects distributed at surface regions. We further employ effective passivation agent with functional chemical groups to facilitate the healing of the mobile defects, thereby enhancing the V_OC_ to 1.35 V and champion efficiency of 19.38% for WBG perovskite solar cells with a bandgap of 1.81 eV. Subsequently, we develop solution‐processed graphene oxide layer ICLs for tandem application, which not only reduces electrical losses but also improves tandem device stability. The synergistic integration of these two strategies has enabled POTSCs to surpass 25% efficiency while simultaneously achieving enhanced operational stability.

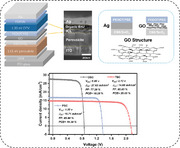

## 1 Introduction

The global demand for sustainable and renewable energy sources has intensified research into advanced photovoltaic technologies. Tandem solar cells (TSCs), which stack multiple layers of different photovoltaic materials, offer a promising route to exceed the Shockley–Queisser limit of single‐junction solar cells by efficiently utilizing a broader spectrum of sunlight.^[^ ^1^ ^]^ Among the various tandem configurations, perovskite‐organic tandem solar cells(POTSCs) have emerged as a promising candidate due to the complementary properties of perovskite and organic semiconductor materials. Both materials offer advantages such as tunable bandgaps, low‐temperature solution processing, and the potential for lightweight and flexible applications, making them ideal for next‐generation thin‐film photovoltaic technologies.^[^ ^2^, ^3^ ^]^

Despite their potential, the development of efficient and stable POTSCs is hindered by several challenges. The power conversion efficiency (PCE) of POTSCs is relatively lower than that of other perovskite‐based TSCs, including perovskite‐silicon TSCs and all‐perovskite TSCs.^[^ ^4^, ^5^, ^6^, ^7^, ^8^ ^]^ A primary challenge in POTSCs is the requirement for wider bandgap perovskite subcells to achieve current matching. The wider bandgap requirement consequently leads to more pronounced *V*_OC_ losses in the wide‐bandgap (WBG) perovskite solar cell (PSC) as the top subcell in POTSCs.^[^ ^9^ ^]^ For WBG perovskites with a bandgap of over 1.8 eV, a high ratio of bromide (Br) is necessary to match the current with organic subcells in POTSCs. However, a high Br content leads to faster crystallization of the WBG perovskite film.^[^ ^10^ ^]^ The uncontrollable crystallization kinetics inevitably contribute to defect formation, manifesting as structural and compositional heterogeneity, along with a high defect density at grain boundaries and surfaces.^[^ ^11^ ^]^ To tackle these issues, diverse strategies—including crystallization optimization, composition regulation, additive engineering, and interface modification—have been developed to enhance the properties of WBG perovskites, thereby improving the PCE of corresponding WBG PSCs.^[^ ^12^, ^13^, ^14^, ^15^, ^16^ ^]^

In addition, optimizing the interconnecting layer (ICL) is critical for POTSC performance, as it governs the electrical, optical, and mechanical coupling between front and rear subcells. For high‐performance POTSCs, two primary options for ICLs are sputtered transparent conductive oxide (TCO)‐based ICLs and ultrathin thermally evaporated metal‐based ICLs.^[^ ^17^, ^18^, ^19^, ^20^, ^21^, ^22^, ^23^, ^24^, ^25^, ^26^ ^]^ Nevertheless, significant limitations persist in both approaches. Sputtered TCO‐based ICLs can cause plasma‐induced damage to underlying perovskite layers without protective buffer interlayers. Metal‐based ICLs exhibit a fundamental thickness‐dependent compromise between charge transport efficiency and optical transmittance. Also, metal ion interdiffusion into perovskite absorbers may accelerate TSC degradation.^[^ ^27^, ^28^, ^29^ ^]^ To address this, M. Stolterfoht et al. reported ultrathin ALD‐InOx ICL to minimize *J_SC_* loss.^[^ ^30^ ^]^ Besides this, solution‐processed ICLs are barely reported in perovskite tandem solar cells. Therefore, it's eager to develop solution‐processed and low‐cost ICLs to achieve reliable, efficient, and stable POTSCs.

In this work, we demonstrate a synergistic strategy to simultaneously enhance the efficiency and stability of POTSCs. Our approach involves two critical innovations: i) mitigating mobile deep defects at the WBG perovskite surface, and ii) implementation of solution‐processed graphene oxide (GO) ICLs with enhanced stability and efficient charge recombination property. Retrospective analysis identifies mobile defects near the surface region as the primary source of energy losses in WBG PSCs. We passivate these mobile defects via bifunctional molecular modulation using 2‐(piperazine‐1‐yl) ethylamine hydroiodide (PipEAI). This defect engineering yields WBG PSCs (E_g_ = 1.81 eV) with a PCE of 19.38% and a *V*_OC_ of 1.35 V. Subsequent integration of optimized GO‐based ICLs into POTSCs further enhances the tandem device PCE to over 25%. Importantly, the GO‐based POTSCs exhibit good operational stability, retaining 92.2% of its initial efficiency under continuous maximum power point tracking (MPPT) over 500 h.

## 2 Results and Discussion

### 2.1 Mobile Defects Mitigation in WBG PSCs

**Figure 1:**
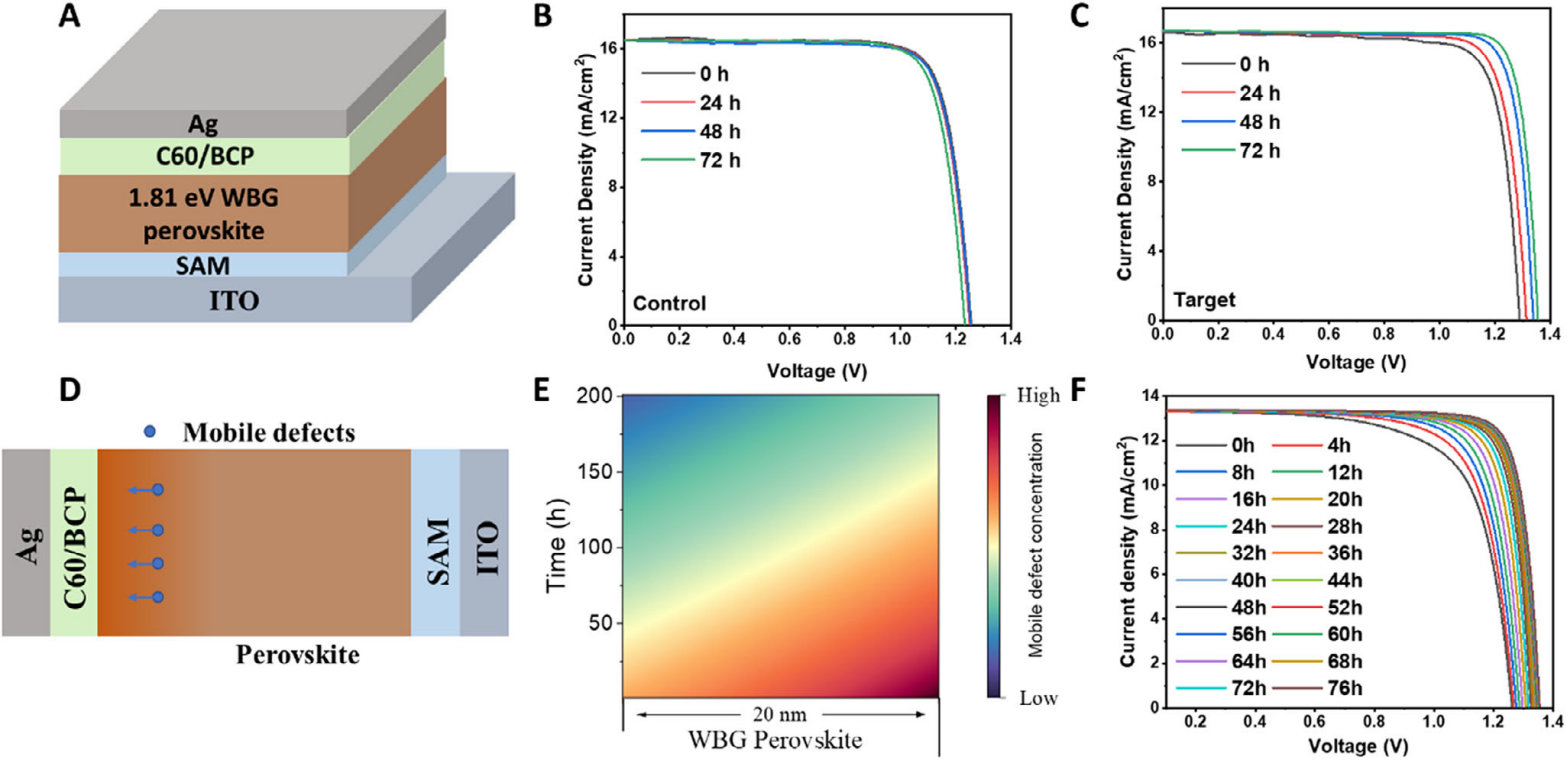
Mitigation of mobile defects in WBG PSCs. A) Structure of WBG PSCs. B,C) *J‐V* curves of the control and target WBG PSCs after different times of room‐light aging. D) Schematic diagram showing the defects translated into the perovskite layer. E) A Simulation diagram illustrates the evolution of defect distribution over time and distance across the top surface region of the WBG perovskite region. F) Simulated *J‐V* curves of the target WBG PSC changes over time.

One of the most significant obstacles facing WBG PSCs is large *V*_OC_ loss, which will reduce the overall efficiency and restrict their application in efficient POTSCs. One potential explanation for this phenomenon is the distinctive defect activity observed in WBG PSCs.^[^ ^31^, ^32^ ^]^ While strategies like passivation and enhancing crystallinity can reduce defect density, the thermodynamic properties of these unfavorable defects are still not well understood. Moreover, most point defects in perovskite materials are mobile. This mobility causes the spatial distribution of these defects to change over time during device operation or device aging.^[^ ^33^ ^]^ As a result, these changes can significantly impact the overall device performance. **Figure** **1**A illustrates the structural configuration of WBG PSCs with a p‐i‐n structure: ITO/self‐assembled monolayer (SAM)/Cs_0.2_FA_0.8_Pb(I_0.6_Br_0.4_)_3_/C_60_/BCP/Ag, where ITO is indium tin oxide, SAM is a self‐assembled layer (4PADCB), C_60_ is fullerene, and BCP is bathocuproine. The detailed device fabrication process was provided in the experimental section. Figure 1B shows the typical current density‐voltage (*J‐V*) characteristics of the WBG perovskite devices with different aging times under room light. It is important to note that no modifications or optimizations, such as surface passivation, were applied to the WBG PSCs (denoted as Control WBG PSCs). The Control WBG PSCs exhibit an initial efficiency of 16.64%, with a *V*_OC_ of 1.256 V, fill factor (FF) of 80.30%, and short‐circuit current density (*J*_SC_) of 16.50 mA cm^−2^. After a 72‐h aging process, the PCE experienced a slight decline to 16.22%, accompanied by a *V*_OC_ of 1.232 V, a FF of 79.82%, and a *J*_SC_ of 16.49 mA cm^−2^ (Table S1, Supporting Information). However, the *V*_OC_ deficit for WBG perovskite devices remains considerable, with an average *V*_OC_ deficit of 0.56 V tested across the devices with different aging times. Surface passivation approaches have been extensively employed to eliminate the surface defects, thereby suppressing non‐radiative recombination and improving *V*_OC_.^[^ ^34^, ^35^ ^]^ WBG PSCs with four different surface passivation materials are investigated, and their results and statistics data are shown in Figures S1 and S2, Table S2 (Supporting Information). Among them, PipEAI demonstrates the highest passivation capability, likely due to its multifunctional groups. Unlike conventional ammonium salts, PipEAI have an additional R2NH group to format hydrogen bonds N─H···N with the A‐site organic cations and replace a halide to bond with Pb to form a Pb−N bond to suppress the ion migration. The NH and NH_3_⁺ groups effectively passivate surface defects and suppress nonradiative recombination (see Figure S3, Supporting Information for chemical structure).^[^ ^36^ ^]^). Here, WBG PSCs with PipEAI as a passivation agent is denoted as Target WBG PSCs. As depicted in Figure 1C, the *J‐V* curves of Target WBG PSCs were presented with various aging times. Compared to the control devices in Figure 1B, the Target devices exhibit a slight improvement in the initial PCE, with the *V*_OC_ increasing from 1.256 to 1.289 V. Furthermore, as aging progresses, the *V*_OC_ of the surface‐passivated WBG perovskite devices significantly rises from 1.289 to 1.355 V, and maintains this level for a prolonged period (Figure S4, Supporting Information), resulting in an enhanced PCE from 17.15% to 19.38% (Table S3, Supporting Information). Figure S5 (Supporting Information) shows the target device *J‐V* test after 72 h of aging, under dark conditions, the device can maintain its best performance for a long time. Moreover, the target device exhibited negligible hysteresis than the control device (Figure S6, Supporting Information). Detailed parameters of the devices are shown in Tables S4 and S5 (Supporting Information). The statistical performance of 20 target PSCs before and after 72 h of aging is presented in Figure S7 (Supporting Information), indicating a universal improvement in WBG devices following the aging process. This observation can likely be attributed to the migration of mobile defects toward the surface region, where they are subsequently passivated by the passivation agents. As illustrated in Figure 1D, we have depicted the migration process of point defects in WBG PSCs near the top surface region. To simulate the defect migration process in target WBG PSCs, an optoelectronic simulation of a WBG PSC was conducted, accounting for the gradual migration of mobile defects over time from the bulk to the top surface. Once these mobile defects reach the top surface, they are effectively mitigated through interaction with the passivation molecules. Detailed simulation methods and parameters are provided in the simulation section. The simulation results, illustrated in Figure 1E, show the concentration of mobile defects at different locations over time. Additionally, Figure 1F presents the simulated *J‐V* curves of the corresponding WBG PSCs. These results clearly demonstrate a gradual improvement in device performance over time, consistent with the experimental observations presented in Figure 1C.

**Figure 2:**
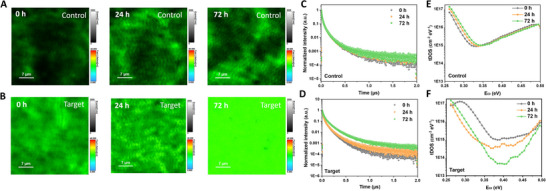
Optoelectronic characterizations of WBG perovskite films and devices. (A and B) PL mapping images of the control and target perovskite films with different aging times. (C and D) TRPL decay curves of control and target perovskite films with different aging times. (E and F) t‐DOS of the control WBG PSC and the target WBG PSC with different aging times.

**Figure 3:**
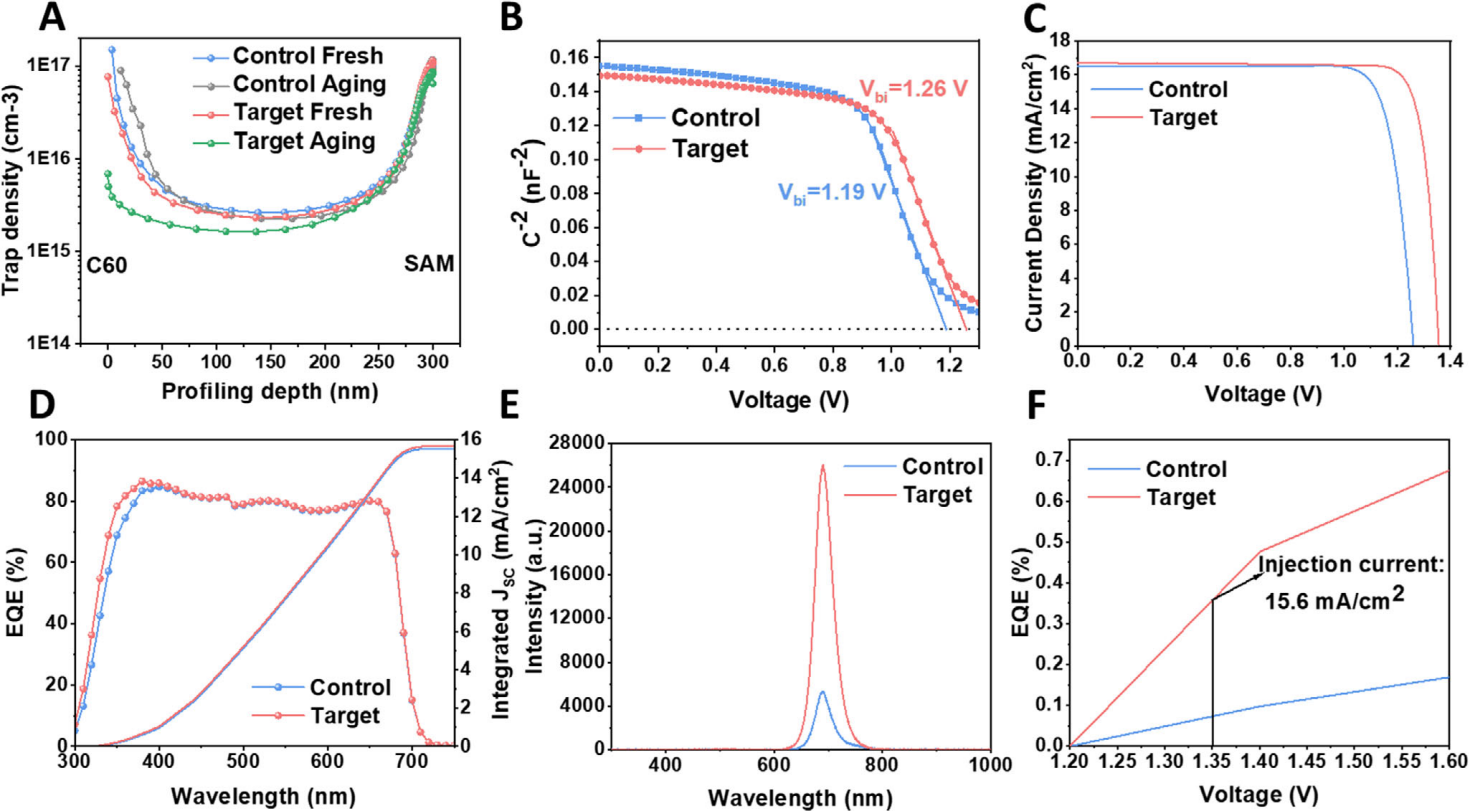
Photovoltaic performance of WBG PSCs. A) Dependence of the trap density on the profiling distance for Control and Target WBG PSC. B–D) (B) Mott–Schottky curves, C) Best *J‐V* curves, D) EQE and integrated *J_SC_* of the control and target WBG PSCs. E) EL intensity of control and target WBG PSCs. F) EQE_EL_ of control and target WBG PSCs with different voltage bias. (All of the control and target WBG PSCs were tested after 72 h aging).

To validate our hypothesis that the enhancement in device efficiency is driven by the mitigation of mobile defects during the aging process, we conducted a series of characterizations. We first examined the evolution of Target WBG perovskite film qualities, including morphology and crystallinity, during the aging process. No significant differences in the Target WBG perovskite film morphology were observed after the aging process, as demonstrated by the top‐view scanning electron microscopy (SEM) images in Figure S8 (Supporting Information). Subsequently, the crystallinity of Target WBG perovskite films was evaluated after the aging process. The X‐ray diffraction (XRD) patterns in Figure S9 (Supporting Information) reveals no significant differences between the fresh and aged films. Therefore, it can be concluded that enhanced performance is not ascribed to the improved film morphology and crystallinity resulting from the aging process. We further investigated the optoelectronic properties of WBG perovskite films and devices. **Figure** **2**A,B, and S10 (Supporting Information) illustrates the evolution of photoluminescence (PL) mapping images for the control and target samples throughout the aging process. The PL intensity and lifetime of the control film remains nearly unchanged after aging, as shown in Figure 2A. In contrast, the target film exhibits a significant increase in PL intensity after aging (Figure 2B; Figure S9, Supporting Information), suggesting that mobile defects are gradually mitigated during the aging process. To further investigate the defect mitigation effect, time‐resolved photoluminescence (TPRL) spectroscopy was employed (Figure 2C,D). The target film shows an enhanced PL lifetime after aging, reinforcing the conclusion that mobile defects are being effectively passivated. Despite of *V_OC_* loss, ion migration has an impact on device *J_SC_*.^[^ ^37^ ^]^ To evaluate this, we did the J‐V test at different scan speed (Figure S11, Supporting Information). Compared to the target device, the control device exhibits severe *J_SC_* loss at slow scan speed, corresponding to the increased concentration of mobile ions. Additionally, thermal admittance spectroscopy, a well‐established method for quantifying the trap density of states (t‐DOS) in thin‐film photovoltaics, was conducted. As illustrated in Figure 2E, the control device exhibits a slight increase in shallow defect densities and a minor reduction in deep trap densities after aging. In contrast, the target WBG PSC demonstrates a significant decrease in trap state density following the aging process (Figure 2F). This trend correlates with the observed improvements in *V*_OC_ and efficiency, further reinforcing the role of mobile defect mitigation in enhancing the performance of the target devices. To further elucidate the spatial mitigation of trap density in WBG PSCs before and after aging, we performed the drive‐level capacitance profiling (DLCP) measurement. This technique allows for a detailed analysis of the spatial distribution of trap states throughout the device.^[^ ^38^ ^]^ As shown in Figure 3A, the aged target WBG PSC exhibits a significantly reduced trap density compared to the fresh one. By contrast, no discernible changes are observed in the control device before and after the aging. The DLCP profiling reveals that the trap density near the C_60_/perovskite interface is approximately one order of magnitude lower in the target devices after aging, further verifying our hypothesis.

We then conducted a series of characterizations, which further confirmed that the aged target WBG PSCs exhibited less trap density than the control WBG PSCs. The Mott‐Schottky plot in **Figure** **3**B indicates that the built‐in potential (*V*_bi_) of the target WBG PSC (1.26 V) is higher than that of the control one (1.19 V). This increase in *V*_bi_ suggests a reduction in defect state density and an improvement in the efficiency of carrier transport within the target WBG PSCs. The *J‐V* curves of the champion control and target WBG PSCs are displayed in Figure 3C. The champion control WBG PSC demonstrates a comparatively inferior PCE of 16.64%, with a *V_OC_* = 1.256 V, *J_SC_* = 16.50 mA cm^−2^, and FF = 80.30%. In contrast, the best‐performing target WBG PSC yielded an optimal efficiency of 19.38% and a steady‐state PCE of 19.32% (Figure S12, Supporting Information). The integrated *J*_SC_ values for the control and target PSCs are 16.05 and 16.15 mA cm^−2^, derived from the external quantum efficiency (EQE) spectra, closely match each other, and align with the *J*_SC_ values obtained from the *J‐V* curves. The bandgap, obtained from the EQE data (Figure 3D), is determined to be 1.81 eV. The primary enhancement arises from a significant increase in the *V_OC_*. It is noteworthy that the champion device generates a high *V_OC_* of 1.35 V, corresponding to a very low *V_OC_* loss of 460 mV. Furthermore, electroluminescence (EL) measurements were conducted to quantify the nonradiative *V_OC_* loss in WBG PSCs, providing further investigation into defect‐related losses. As shown in Figure 3E and Figure S13 (Supporting Information), under identical injection current conditions, the target WBG PSC exhibits an EL intensity that is over five times higher than that of the control one, indicating the reduced nonradiative recombination losses. We further measured the external quantum efficiency of electroluminescence (EQE_EL_) of control and target WBG PSCs. As shown in Figure 3F, the target WBG PSC yields an EQE_EL_ of 0.36% when the injection current is equivalent to the *J_SC_*. In contrast, a considerably low EQE_EL_ of 0.02% was obtained for the control WBG PSC. The two‐order‐of‐magnitude enhancement in EQE_EL_ value substantiates the markedly diminished nonradiative recombination losses, corroborating the enhanced *V*_OC_ observed in the target WBG PSC.

### 2.2 Photovoltaic Performance of POTSCs with GO ICLs

Having demonstrated high‐performance WBG PSCs via the mitigation of mobile defects, we turned our focus to the design of POTSCs. ICL is a critical component in TSCs. Here, we proposed to use solution‐processed GO as the ICL to replace the conventional thermally evaporated thin metal film, leveraging its excellent optical, electrical, and optoelectronic properties.^[^ ^39^ ^]^ GO's unique characteristics, such as high transparency, tunable conductivity, make it an ideal candidate for effective ICL in TSCs. Recently, the GO layer has been reported to perform effectively well as an ICLs in all‐perovskite TSCs.^[^ ^40^ ^]^ In this study, 0.2, 0.35, 0.5, and 1 mg mL^−1^ GO in deionized water were prepared (Figure S14, Supporting Information). GO exhibits very good dispersion in deionized water with varying concentrations. To examine the morphology of GO thin films, we spin‐coated GO layers onto silicon substrates and annealed them at 100 °C for 5 min. As shown in the zoomed‐in SEM images in Figure S15 (Supporting Information), the layered structure of GO is clearly visible, confirming the formation of 2D GO layers. The lateral dimensions of the GO flakes range from a few hundred nanometers to several micrometers. The GO thin film with isolated features effectively suppresses shunting when employed as an interconnecting layer (ICL) in tandem devices, owing to its high sheet resistance and limited lateral conductivity. Simultaneously, it also promotes efficient vertical charge recombination across the layered GO flakes. To evaluate the optical properties of solution‐processed GO thin films on glass substrates, we conducted UV–vis spectroscopy measurements. The transmittance of solution‐processed GO thin films was examined as a function of wavelength. Figure S16 (Supporting Information) presents the optical transmittance spectra for GO films at varying concentrations, along with a reference 1 nm thermally evaporated Ag thin film. It is observed that the GO films exhibited higher transmission across the 400–700 nm wavelength range.

**Figure 4:**
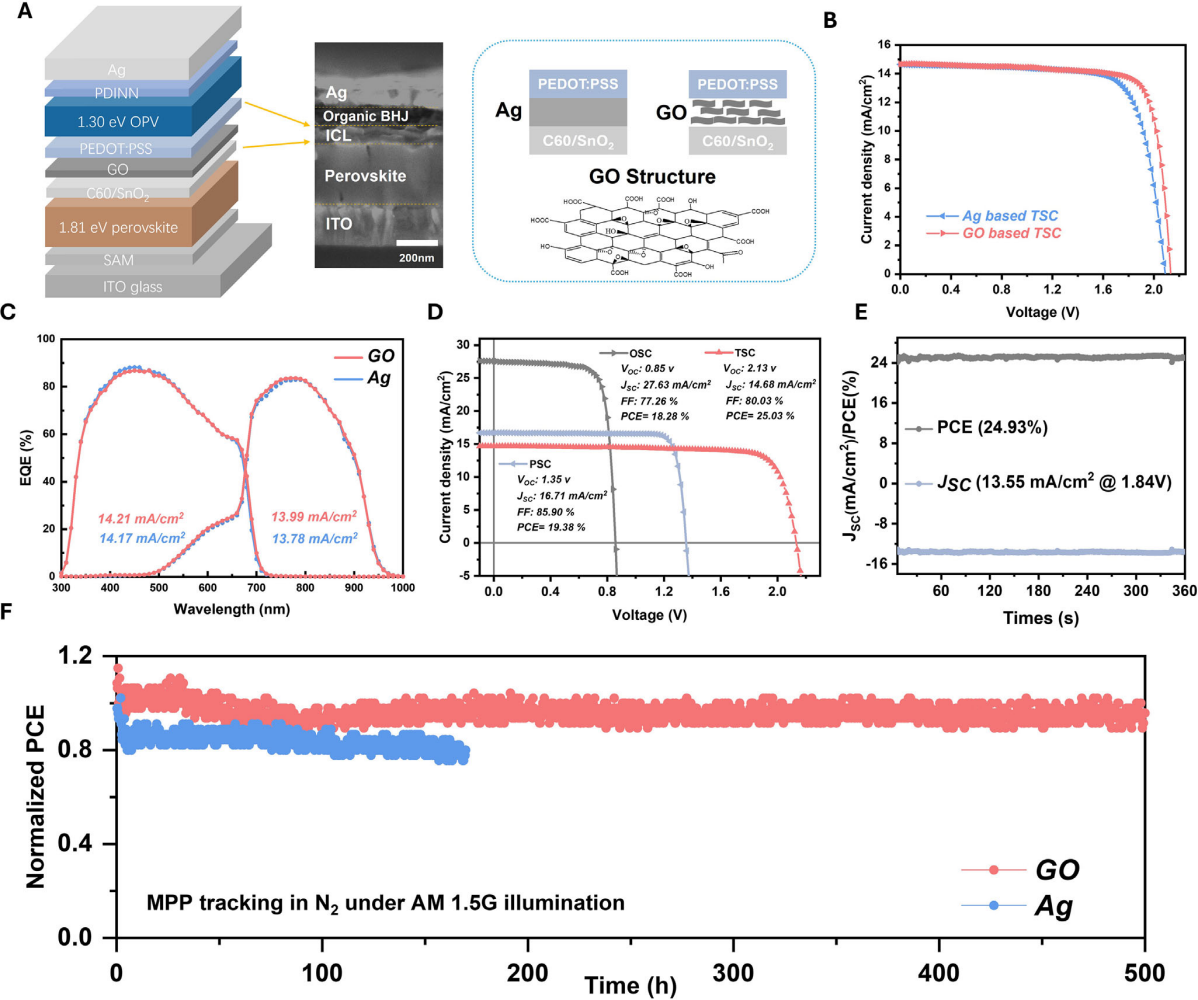
Photovoltaic performance of TSCs. A) Schematic diagram of POTSC and its corresponding cross‐sectional SEM image. B,C) B) *J–V* curves and C) EQE spectra of the champion POTSCs with Ag‐based and GO‐based ICLs. D) Champion efficiency for WBG PSC, NBG OPV, and POTSC. E) The stabilized PCE of GO‐POTSC under MPP and it stabilized *J*_SC_ for 360 s. F) Photostability of the unencapsulated Control and Target POTSC under continuous illumination at the MPP condition in an N_2_ atmosphere.

We then applied the Target WBG PSCs, supplemented by GO ICL, to fabricate monolithic POTSCs. For the organic photovoltaic (OPV) part, we choose PM6:L8‐BO:BTP‐ec9 as the absorption layer and perylene‐diimide (PDINN) as electron transport layer (ETL).^[^ ^41^, ^42^ ^]^ The *J‐V* curve and EQE spectra of single‐junction OPV were shown in Figure S17 (Supporting Information). The OPV device exhibited a *V*_OC_ of 0.85 V, an FF of 77.26%, *J*_SC_ of 27.63 mA cm^−2^, and a PCE of 18.28%. To illustrate the effect of ICLs on the efficiency and stability of POTSCs, we fabricated two types of POTSCs with the configurations of ITO/SAM/WBG perovskite/C_60_/SnO_x_/Ag/PEDOT:PSS/PM6:L8‐BO:BTP‐ec9/PDINN/Ag (denoted as control POTSC) and ITO/SAM/WBG perovskite/C_60_/SnO_x_/GO/PEDOT:PSS/PM6:L8‐BO:BTP‐ec9/PDINN/Ag (denoted as target POTSC). ALD SnOx is used to replace BCP to prevent the GO water solution's invasion. The target device structure is schematically illustrated in **Figure** **4**A. The cross‐sectional SEM image for the Target POTSC was presented in Figure 4A, corresponding to the schematic diagram. The effect of GO's concentration on the overall efficiency was investigated, as shown in Figure S18 (Supporting Information). The optimal GO concentration was found to be 0.35 mg mL^−1^, which contributes to the highest FF, *V*_OC_, and PCE. We then evaluated and compared the device performance of POTSCs employing either Ag or GO as ICLs. Figure 4B presents a comparative analysis of the *J‐V* curves of control and target POTSCs, respectively. The best‐performing Ag‐based POTSC achieved a PCE of 23.76%, with a *J*_SC_ of 14.65 mA cm^−2^, an FF of 76.21%, and a *V*_OC_ of 2.128 V. By contrast, 0.35 mg mL^−1^ GO‐based POTSC obtained a champion efficiency of 25.03%, along with a *J*_SC_ of 14.67 mA cm^−2^, an FF of 80.16%, and a *V*_OC_ of 2.131 V. Notably, the GO‐based POTSC exhibited an improved average FF (72.96% to 79.01%), enhanced average *V*_OC_ (2.072 to 2.129 V), and better batch‐to‐batch reproducibility, as demonstrated by the performance distribution of 20 devices in Figure S19 (Supporting Information). The observed improvements in *V*_OC_ and FF for GO‐based POTSCs can be ascribed to the enhanced hole extraction capabilities, balanced charge carrier recombination, and minimized nonradiative recombination, as GO can effectively act as a hole transport material.^[^ ^43^ ^]^ The EQE spectrum of the Ag and GO‐based POTSCs were shown in Figure 4C, respectively. For Ag‐based POTSC, the integrated *J*_SC_ values from EQE spectra of perovskite and organic subcells are 14.17 and 13.78 mA cm^−2^, respectively. In contrast, GO‐based POTSC exhibits slightly higher integrated *J*_SC_ values of 14.21 mA cm^−2^ (perovskite subcell) and 13.99 mA cm^−2^ (organic subcell), indicating improved current matching compared to Ag‐based POTSC. The champion efficiencies for optimized WBG PSC, NBG OPV, and GO‐based POTSC are depicted in Figure 4D. As shown in Figure 4E, we assessed the steady‐state PCE and stabilized *J*_SC_ for 360s. The champion POTSC achieves a stabilized PCE of 25.03% with negligible hysteresis (Figure S20, Supporting Information). To assess the impact of ICLs on the operational stability of POTSCs, we subjected unencapsulated POTSCs to continuous 1‐sun illumination under N_2_ atmosphere and monitored their long‐term operation performance. As demonstrated in Figure 4F, the Ag‐based POTSC showed significant performance degradation, retaining only 80% of their initial PCE after 150 h of operation. In contrast, the GO‐based POTSC maintained 92.2% of their initial PCE even after 500 h. The enhanced operational stability may originate from suppressed metal ion diffusion into the WBG perovskite layers through the incorporation of GO ICLs.^[^ ^44^ ^]^ The simultaneous enhancement of both operational stability and efficiency in optimized POTSCs with GO ICLs is crucial for developing high‐performance, stable, and commercially viable perovskite TSCs.

## 3 Conclusion

Our study demonstrates significant advancements in POTSCs, specifically targeting the passivation of mobile defects and the implementation of solution‐processed ICLs. We identify mobile defects located near the top surface as a major contributor to efficiency loss in WBG PSCs. Through post‐treatment and aging methodologies, we effectively eliminate these mobile defects, leading to enhanced performance in WBG PSCs and achieving a champion efficiency of 19.38% with a *V*_OC_ of 1.35 V, corresponding to a low *V*_OC_ loss of 460 mV. Additionally, we introduce solution‐processed GO as ICL, enabling a low‐cost fabrication route for POTSCs while improving charge carrier transport and overall efficiency. As a result, the GO‐based POTSCs deliver a stabilized PCE of 25.03% with good reproducibility. Moreover, compared to the thermally evaporated Ag thin film as ICL, GO‐based POTSC can retain 92.20% of its initial PCE after 500 h of continuous operation under MPP. These innovations represent a significant advancement in the pursuit of high‐performance, stable, and commercially viable perovskite TSCs.

## 4 Experimental Section

### Materials

Lead iodide (PbI_2_) and Lead thiocyanate (Pb(SCN)_2_) were purchased from Tokyo Chemical Industry Co., Ltd (TCI). Formamidinium iodide (FAI), Formamidinium bromide (FABr) were purchased from GreatCell Solar Limited Company. Lead bromide (PbBr_2_), purchased from Liaoning Youxuan Tech Company. Methylammonium chloride (MACl), cesium iodide (CsI), lead chloride (Pb(Cl)_2_), cesium bromide (CsBr), and 2‐(piperazine‐1‐yl) ethylamine hydroiodide (PipEAI, surface‐passivating agent for perovskite), were purchased from Xi'an Yuri Solar Co., Ltd. Dimethyl sulfoxide (DMSO), N, N‐Dimethylformamide (DMF), chlorobenzene (CB), isopropanol (IPA), 1,8‐Diiodooctane (dio), and methanol (MeOH, purity of 99.90%) were purchased from Sigma Aldrich. PM6, L8‐BO, BTP‐ec9, and PDINN were purchased from Solarmer Materials. Graphene oxide powder (analytical pure single‐layer graphene oxide powder (for university experiments)) was purchased from Alibaba (Suzhou Tanfeng Technology). All the chemicals are used directly without further purification.

### The WBG Single‐Junction Device Fabrication

The etched ITO/glass substrates were sequentially cleaned with Glass cleaning agent, Deionized water, Acetone, and IPA. Then heated over night for further use. Before spin coat the perovskite film, the substrate will be treated in a UV‐ozone cleaner for 15 min. The HTL and perovskite absorption layer was fabricated in an N_2_‐filled glove box. 4PADCB solution (1.0 mg mL^−1^) was used to spin coated on the substrate to form HTL layer, after 10 min annealing, dropping 50 µL perovskite precursor (Cs_0.2_FA_0.8_Pb(I_0.6_Br_0.4_)_3_, 1.2 M, DMF: DMSO = 4:1 with 0.5 mol% Pb(SCN)_2_ on the substrate and then spin coated with 4000–6000 rpm for 30s, Chlorobenzene (200 µL) was dropped on the spinning substrate during the 10s before the procedure ended. Subsequently, the sample was annealed at 100°C for 15 min. PipEAI was dissolved in IPA (1.0 mg mL^−1^) as surface passivation with 5000 rpm for 30s. Then the films were annealed at 100°C for 10 min. The C_60_ (≈ 30 nm), BCP (≈ 6 nm) ETL, and Ag (≈ 100 nm) electrode were thermally evaporated onto the surface of the PipEAI through a shadow mask, and the active area is 0.1 cm^2^.

### The OPV Single‐Junction Device Fabrication

OSCs with a p‐i‐n configuration were based on the device structure of Glass/ITO/PEDOT:PSS/PM6:L8‐BO:BTP‐ec9/PDINN/Ag. First, PEDOT:PSS were statically spin‐coated on the clean ITO substrates at 5000 rpm for 30s and followed by thermal annealing at 150°C for 10 min. Then, PM6:L8‐BO:BTP‐ec9 (1:0.6:0.6, w/w/w, 22 mg mL^−1^ total in CB) solution was dynamically cast onto the PEDOT:PSS layer at 2500 rpm for 30 s and followed by thermal annealing at 100°C for 10 min. After cooling, a PDINN (1 mg mL^−1^ in methanol) transporting layer was dynamically spin‐coated onto the organic BHJ layer at 3000 rpm for 30 s. Finally, 100 nm Ag was thermally evaporated through a metal shadow mask (aperture area 0.1 cm^2^).

### The Preparation of GO Solution

To prepare the solution of GO, the GO with the weights of 0.2, 0.35, 0.5, and 1 mg were dissolved in 1 mL of deionized water. The GO solution needs to be stirred overnight and then ultrasonic 30 min to ensure the well dispersion.

### Deposition of ICL

For control POTSCs, 1 nm Ag were thermally evaporated onto the surface of the SnO_x_. Followed by the spin‐coating of PEDOT:PSS on Ag at 5000 rpm for 30s and thermal annealing at 150°C for 10 min. For target POTSCs, GO solution with the concentration of 0.35, 0.5, 1 mg mL^−1^, was dynamically spin‐coated on SnO_x_ layer at 2000 rpm for 30s. Followed by the spin‐coating of PEDOT:PSS on GO layer at 5000 rpm for 30s and thermal annealing at 150°C for 10 min.

### Tandem Device Fabrication

For POTSCs, BCP was replaced by the 20 nm ALD SnO_x_ at a TDMASn dose rate of 0.08s/cycle, water dose rate of 0.05s/cycle under process temperature of 80 °C, and performed 200 cycles of deposition. After the deposition of the control and target ICL layer, respectively, followed by the dynamically coating of PM6:L8‐BO:BTP‐ec9 solution with the same recipe of OPV single‐junction device fabrication. Then, complete the transport layer deposition and the thermal evaporation of the silver electrode.

### Solar Cell Characterization

*J–V* measurements were carried out using a Keithley 2400 source meter in nitrogen filled glove box. The devices were measured both in reverse scan and forward scan with a 10 mV interval and 10 ms delay time. The steady‐state power output curves were recorded by tracing the current density at bias (voltage at maximum power point) for at least 5 min after *J–V* measurements. Illumination was provided by an SS‐F7‐3A solar simulator (Enli Tech. Co., Ltd., Taiwan) with air mass (AM) 1.5G (global) spectrum and light intensity of 100 mW cm^−2^, which was calibrated by a standard Si diode (a KG‐5 reference cell was used for the measurements of WBG perovskite solar subcells). For perovskite/organic TSCs, the solar simulator spectrum was finely tuned to ensure that spectral mismatch was within 3% for both subcells. During J–V measurement, an optical aperture mask (0.1 cm^2^) was used to verify the accurate cell area. EQE measurements for devices were conducted with an Enli‐Tech EQE measurement system. The light intensity at each wavelength was calibrated with a standard single‐crystal Si photovoltaic cell. For EQE measurement of tandem cells, the WBG perovskite front subcells were measured while saturating the organic bottom subcells with continuous light from a halogen lamp with a 700 nm polarized optical filter, while the organic bottom subcells were measured while saturating the perovskite front subcells with continuous light from a halogen lamp with a 600 nm polarized optical filter. No bias voltage was applied for the EQE measurement of both subcells.

### Device Stability

For MPPT measurements, the devices without encapsulation were kept in a nitrogen filled box and tested at ≈33 °C with MSCLT‐1 Multi‐Channels Solar Cells Stability Test System (Wuhan 91PVKSolar Technology Co. Ltd, China). For the maximum power point tracking tests under illumination, the device was fixed at the maximum power point voltage (V_mpp_).

### Film Characterizations

The top‐view WBG aging film and GO on both Si and BCP substrate surface morphology was tested by Field Emission Scanning Electron Microscope (Tescan MAIA3). UV–vis absorption spectra were measured using a Varian Cary 50 Bio UV–Visible Spectrophotometer (Agilent Technologies, Inc.). Crystalline structure was investigated on a Rigaku SmartLab X‐ray diffractometer with Cu Kα radiation in a step of 0.01° and θ‐2θ scan mode from 5° to 50°. Film morphology of the perovskite was measured using a high‐resolution field emission SEM (TESCAN VEGA3). The perovskite film surface was tested using a MultiMode 8‐HR Atomic Force Microscope (AFM, Bruker) with a tapping amplitude modulation mode. PL‐mapping was PL mapping was conducted with the PicoQuant MicroTime 100 and FluoTime 100 system at room temperature. PicoQuant PDL 828 “Sepia II” multichannel diode laser was used as the laser source, and a 405 nm pulsed laser was used for the measurements. Steady‐state PL and TRPL transient decay spectra were measured using a PL spectrometer (Edinburgh Instruments, FLS920) with the excitation source of 636.2 nm picosecond pulsed diode laser (EPL‐635, ≈5 nJ cm^−2^). The EL EQE spectra were recorded by a LED photoluminescence quantum‐yield measurement system (Enli Tech LQ‐100) equipped with a Keithley 2400 Source Measure Unit.

### Other Characterizations

The t‐DOS and DLCP measurements were both performed by KEYSIGHT E4980A Precision LCR Meter. For DLCP measurement, it was conducted in the direct current (DC) bias range in 20 mV. While the amplitude of the alternative current (AC) biases ranged from 20 to 200 mV.^[^ ^38^ ^]^ While the amplitude of the AC biases ranged from 20 to 200 mV. For each AC bias, an additional offset DC voltage was applied to keep the maximum forward bias constant. The selected test frequencies are 10, 20, 50, 100, 200, and 500 kHz, respectively. The trap density was estimated by subtracting the estimated free carrier density, which was measured at high AC frequencies when the measured carrier densities saturate with the further increase of the AC frequency, from the total carrier density measured at the low ac frequency. This technique allowed us to derive the energetic distribution (Eω) of trap states by tuning the frequency of the AC bias (δV) or temperature (T) and the position of trap states in real space by changing the DC bias that was applied to the depletion region of the junction. For t‐DOS measurement, the DC bias was fixed at 0 V, and the amplitude of the AC bias was 100 mV. The scanning range of the AC frequency was 0.02–2000 kHz. The t‐DOS (NT (Eω)) is calculated by using the equation NT (Eω) = – $\frac{1}{qkT}\;\frac{\omega dC}{d\omega }\frac{Vbi}{W}$, where W and Vbi are the depletion width and build‐in potential, respectively, which were derived from the Mott‐Schottky analysis of the C‐V measurement. q, k, T, ω, and C are the elementary charge, Boltzmann's constant, temperature, angular frequency, and specific capacitance, respectively, which were derived from the Mott‐Schottky analysis of the C‐V measurement. q, k, T, ω, and C are the elementary charge, Boltzmann's constant, temperature, angular frequency, and specific capacitance, respectively.

### Simulation

Using COMSOL Multiphysics, a coupled optoelectronic model was developed based on the finite‐element method, incorporating Maxwell's equations, electron/hole transport equations, cation/anion transport equations, and the Poisson equation.

## Conflict of Interest

The authors declare no conflict of interest.

## Author Contributions

Y.H. and Q.L. contributed equally to this work. G.Y. and G.L. conceived the idea and supervised the project. Y.H. and Q.L. designed and performed the most of the experiments. Y.H. contributed to the fabrication and characterization of perovskite solar cells. Q.L. and J.Y. contributed to fabrication and characterization of organic solar cells. K.J. performed SEM and XRD measurements. Z.A. performed the simulation. F.Z. and Z.N. performed PL‐mapping measurement. Y.Z. and Y.Z. contributed to the optimization of WBG solar cells. Y.C. and Y.B. contributed to the optimization of tandem device. G.Y., Y.H., Q.L. contributed to the writing of this manuscript. Y.B. and G.L. participated in the discussion and commented on the manuscript. All authors contributed to reviewing of the manuscript.

## Supporting information



Supporting Information

## Data Availability

The data that support the findings of this study are available from the corresponding author upon reasonable request.
