# Supplementary material for: Defect Dynamics and Solution‐Processed Interconnects in Perovskite‐Organic Tandem Solar Cells
Source: Adv Sci (Weinh). 2025 Dec 12;13(12):e19528. doi: 10.1002/advs.202519528 (PMC12948246; doi:10.1002/advs.202519528)
Supplement: Supplementary file 1 — Supporting Information [file ADVS-13-e19528-s001.docx]

Supplemental information

**Defect Dynamics and Solution-Processed Interconnects in Perovskite-Organic Tandem Solar Cells**

*Yingjie Hu^1,6^, Qianyi Li^1,6^, Kaifeng Jing^1^, Jiangsheng Yu^1^, Fuyi Zhou^4^, Zhenhai Ai^1^, You Chen^3,5^, Yue Zhao^1^, Yijia Zhang^1^, Zhenyi Ni^4^, Yang Bai^3,5*^, Gang Li^1,2*^, Guang Yang^1,2*^*

^1^Department of Electrical and Electronic Engineering, The Hong Kong Polytechnic University, Hung Hom, Kowloon, Hong Kong, China

^2^Photonic Research Institute (PRI), Research Institute of Smart Energy (RISE), Research Institute for Advanced Manufacturing (RIAM), The Hong Kong Polytechnic University, Hung Hom, Kowloon, Hong Kong, China

^3^Faculty of Materials Science and Energy Engineering, Shenzhen University of Advanced Technology, Shenzhen 518107, China

^4^State Key Laboratory of Silicon and Advanced Semiconductor Materials & School of Materials Science and Engineering, Zhejiang University, Hangzhou 310027, China

^5^Institute of Technology for Carbon Neutrality, Shenzhen Institutes of Advanced Technology (SIAT), Chinese Academy of Sciences, Shenzhen 518055, China.

^6^These authors contributed equally: Yingjie Hu, Qianyi Li

Email: y.bai@siat.ac.cn, gang.w.li@polyu.edu.hk, guang.yg.yang@polyu.edu.hk

Supplemental Data


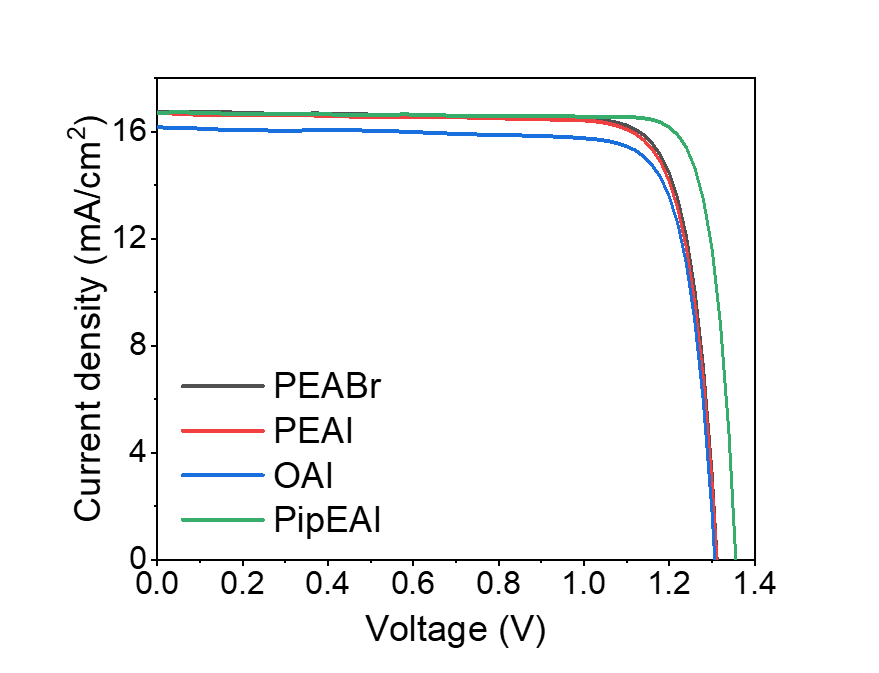


**Figure S1.** *J-V* curves of PSCs with different surface passivation under reverse scan.


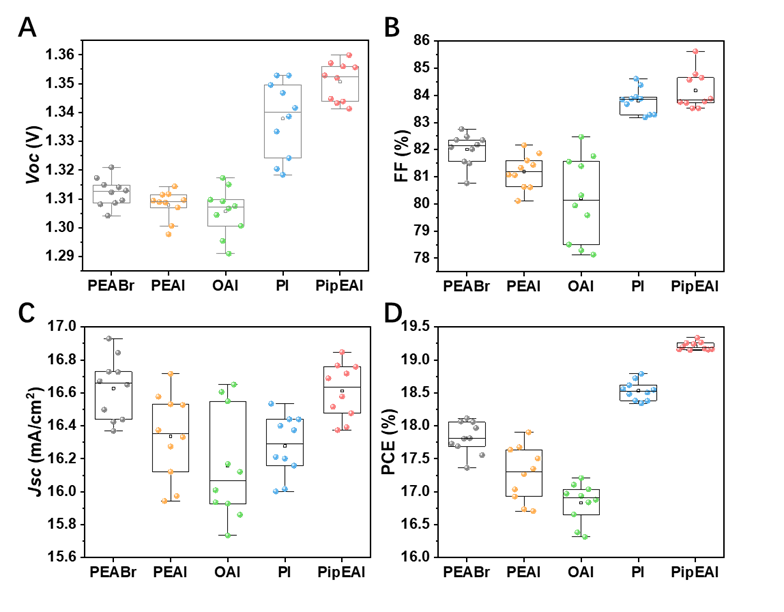


**Figure S2.** Statistics of (A) *V*_OC_, (B) FF (C) *J_SC_* and (D) PCE for 10 WBG PSCs with different surface passivation under reverse scan.


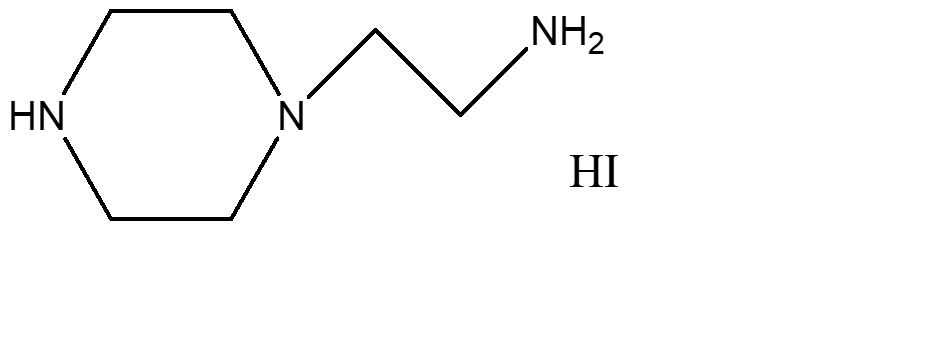


**Figure S3.** The chemical structure of 2-(piperazine-1-yl) ethylamine hydroiodide (PipEAI).


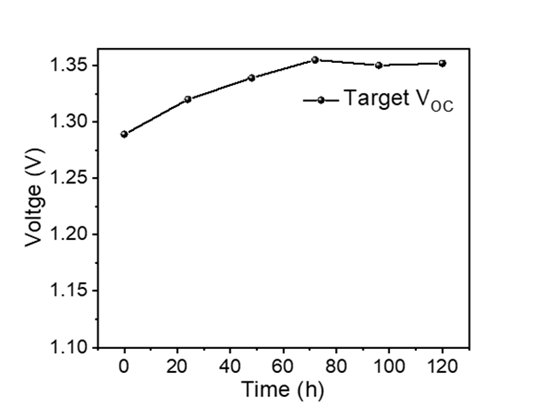


**Figure S4.** The *V_OC_* of the target device with different aging times (device was stored in a nitrogen-filled glove box and in room light and temperature).


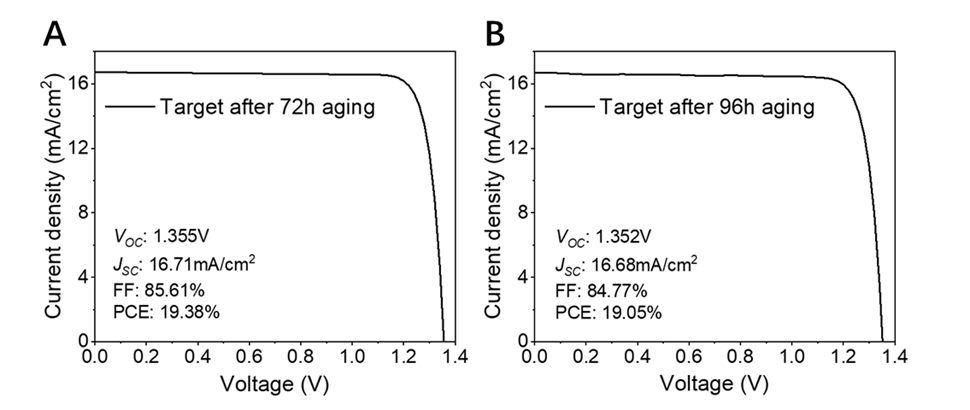


**Figure S5.** The *J-V* curves of the target device after 72h (A) and 96h (B) aging (The device is stored in dark and nitrogen-filled glove box).


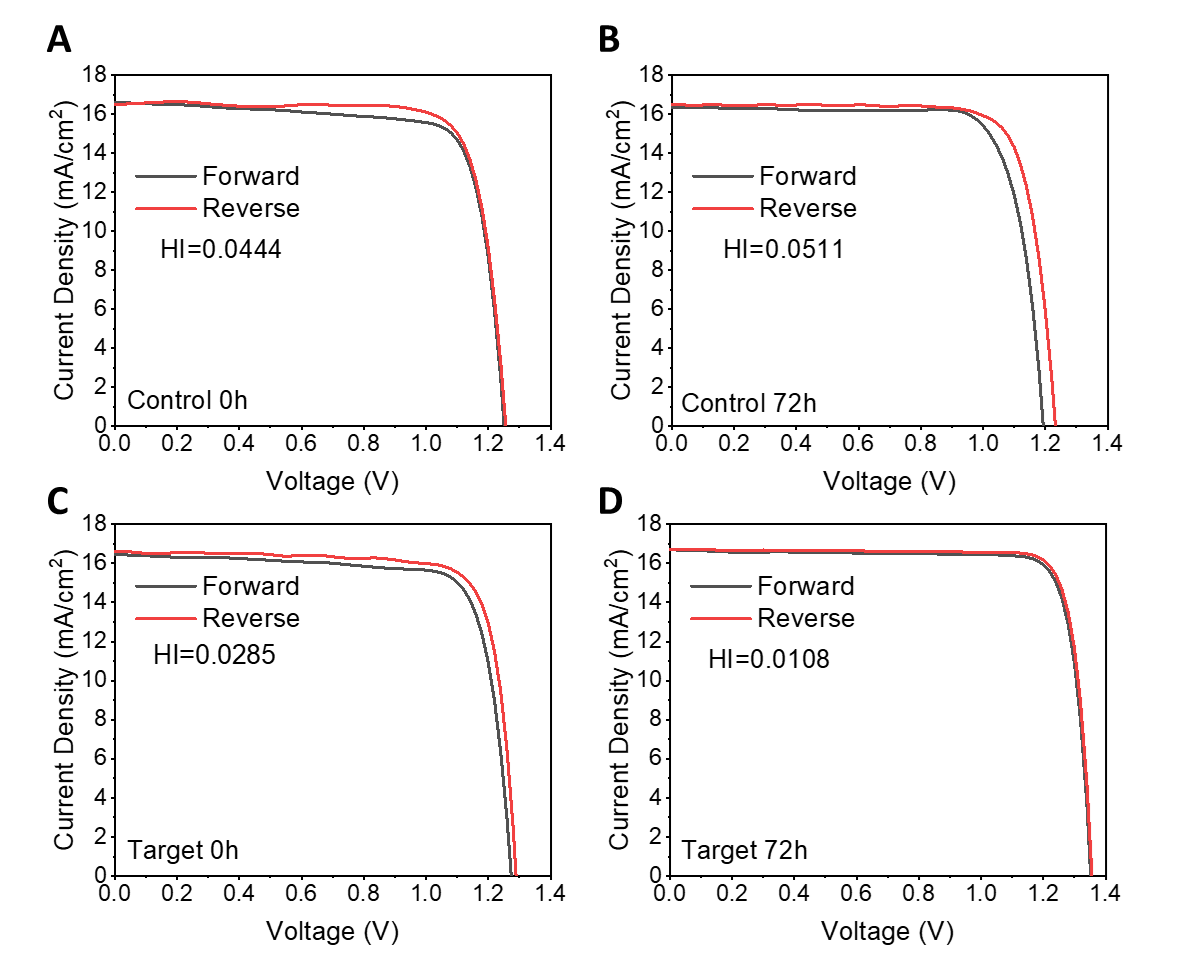


**Figure S6.** Forward and reverse scan of *J-V* curves of control after (A) 0h, (B) 72h aging and target after (C) 0h, (D) 72h aging. (forward, 1.4 to −0.1 V; reverse, −0.1 to 1.4 V). Hysteresis index (HI) was calculated using the equation, HI = [PCE(forward)-PCE(reverse)]/PCE(forward)


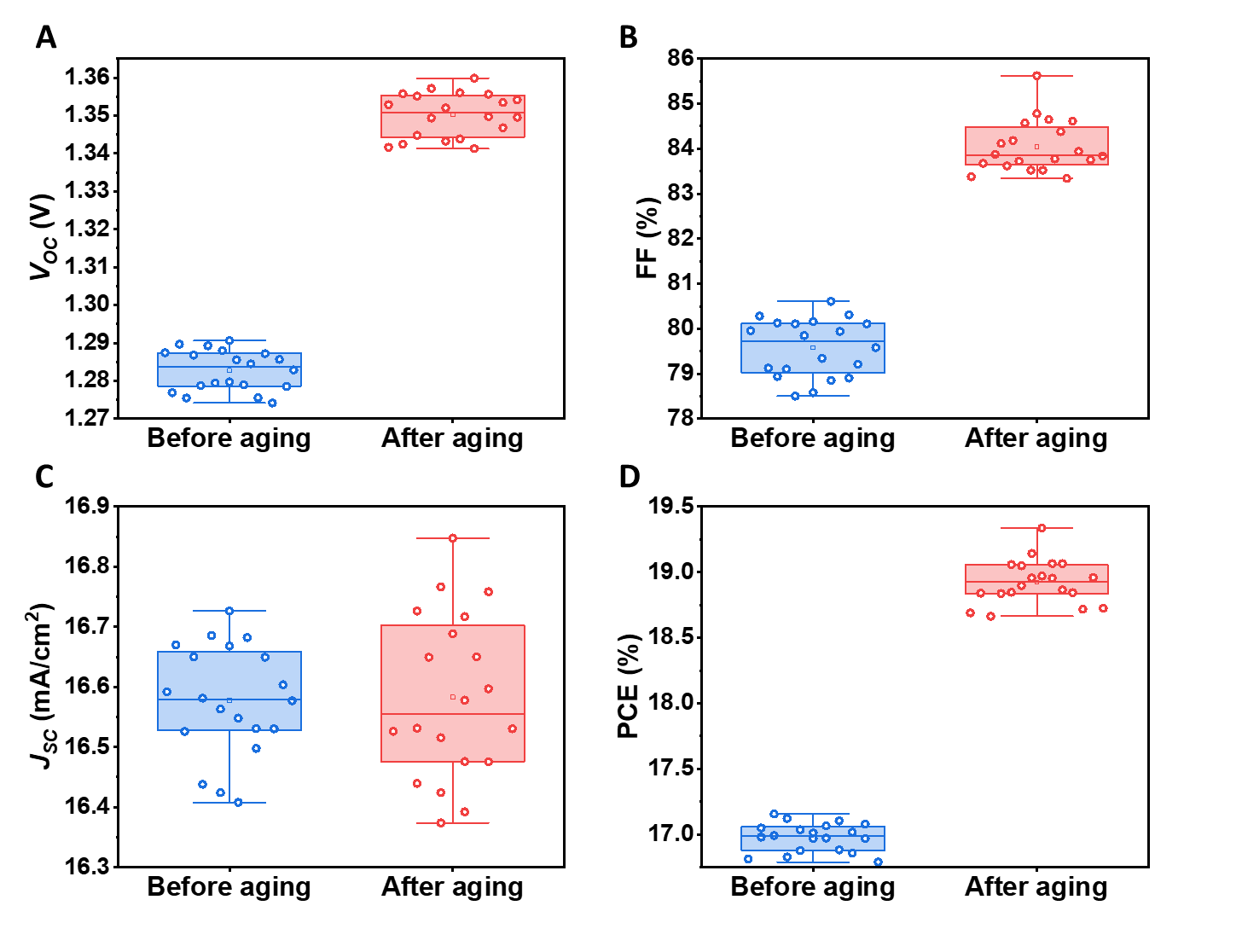


**Figure S7.** Statistics of (A) *V*_OC_, (B) FF (C) *J*_SC_ and (D) PCE for 20 WBG PSCs before and after 72h aging. Statistical parameters were analyzed for both before and after aging devices (aperture area = 0.1 cm²) across 20 devices from four independent batches. The boxplots display: whiskers (data minima and maxima), box boundaries (25th and 75th percentiles), and center line (mean value).


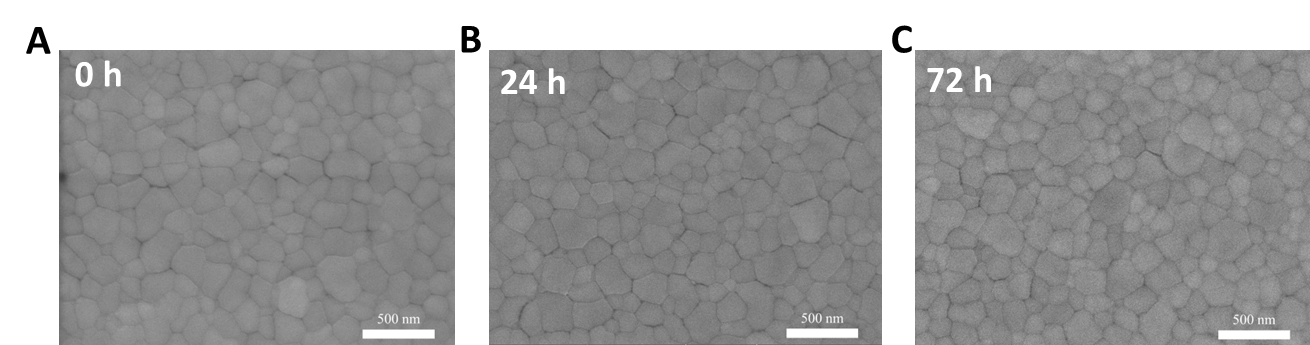


**Figure S8.** Top-view SEM images of Target WBG perovskite films after (A) 0h, (B) 24h and (C) 72h aging.


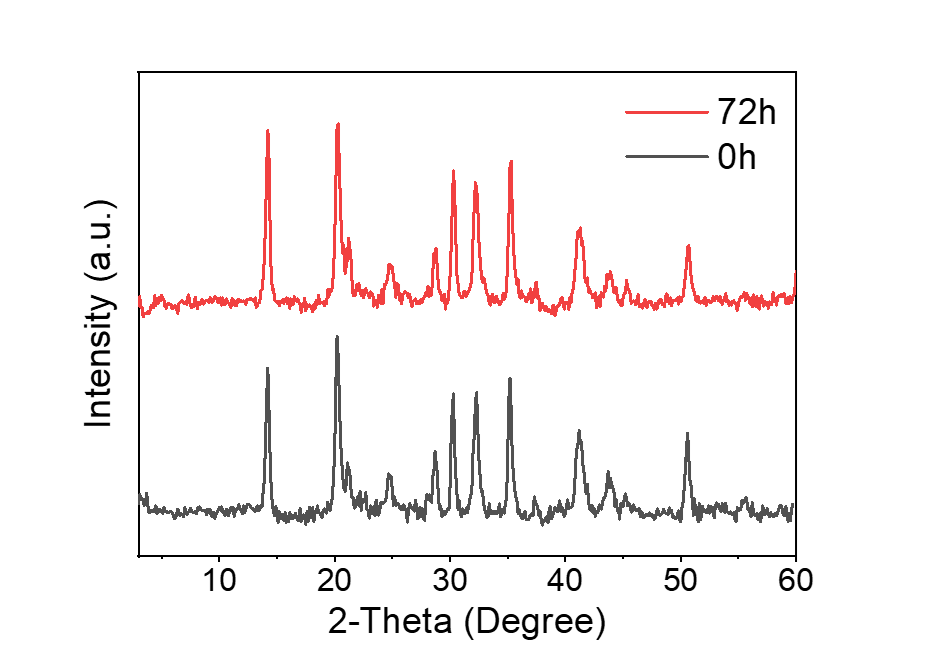


**Figure S9.** XRD patterns of target WBG perovskite films with aging time 0h and 72h.


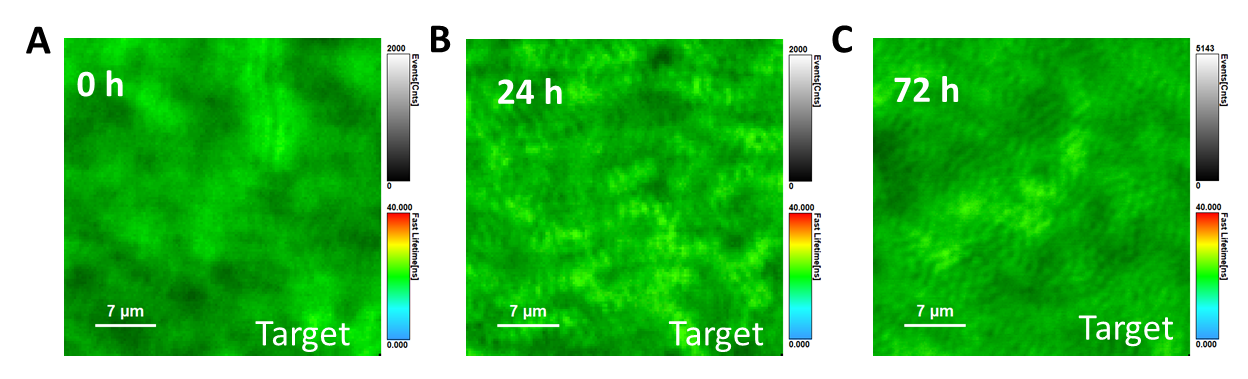


**Figure S10.** PL mapping images of WBG perovskite films with aging time (A) 0h, (B) 24h and (C) 72h. It should be noted that the color bar for counts in Fig. C is distinct from that in Fig. A and B.


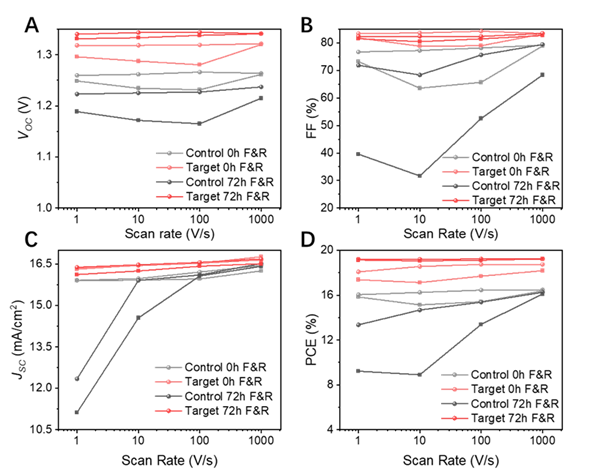


**Figure S11.** The *V_OC_* (A), FF (B), *J_SC_* (C) and PCE (D) of *J–V* test measured at different scan speeds in reverse (R, ball) and forward (F, square) scan directions in different aging times.


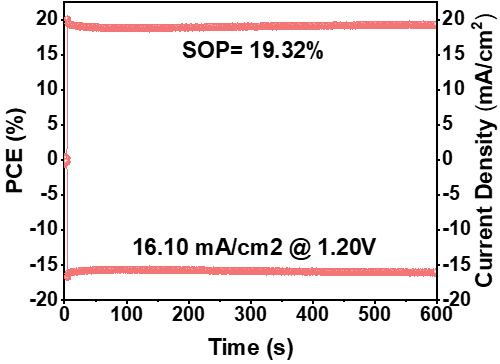


**Figure S12.** Stabilized efficiency of target WBG PSC.


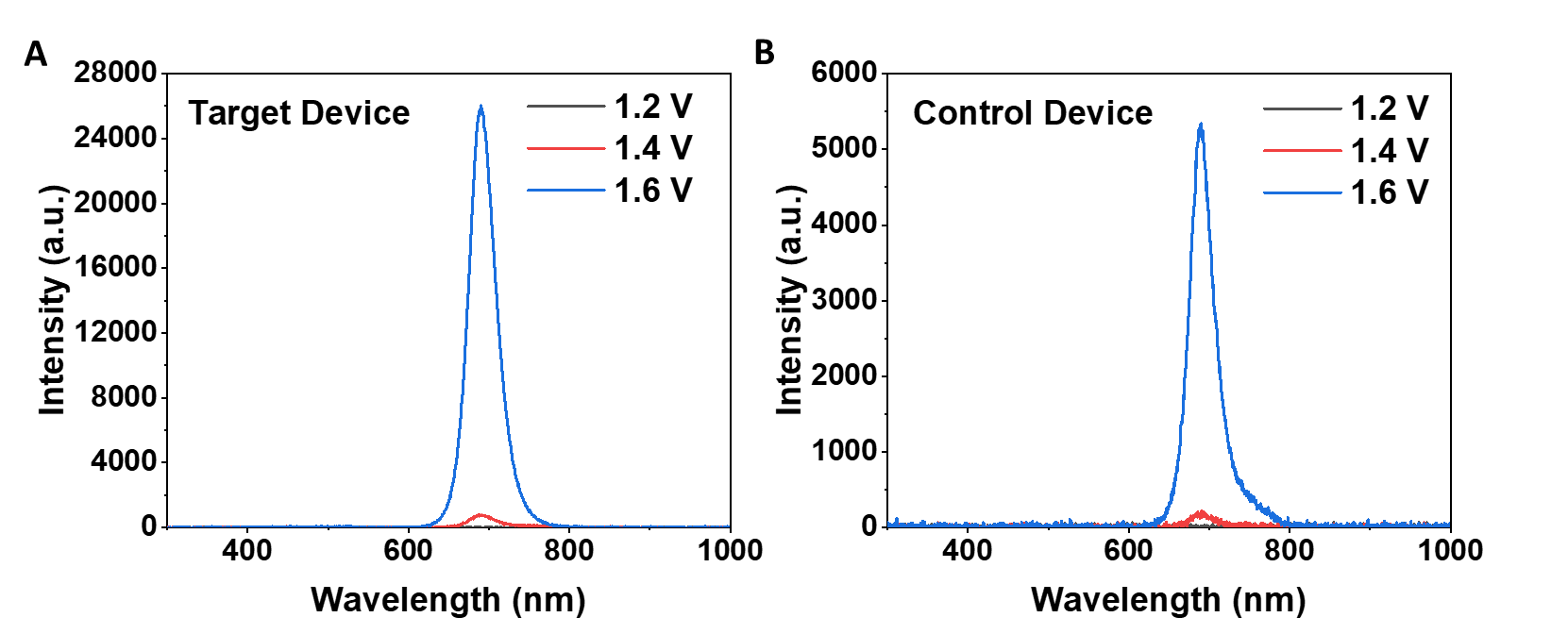


**Figure S13.** EL intensity of (A)Target and (B)Control WBG perovskite films with different injection voltage.


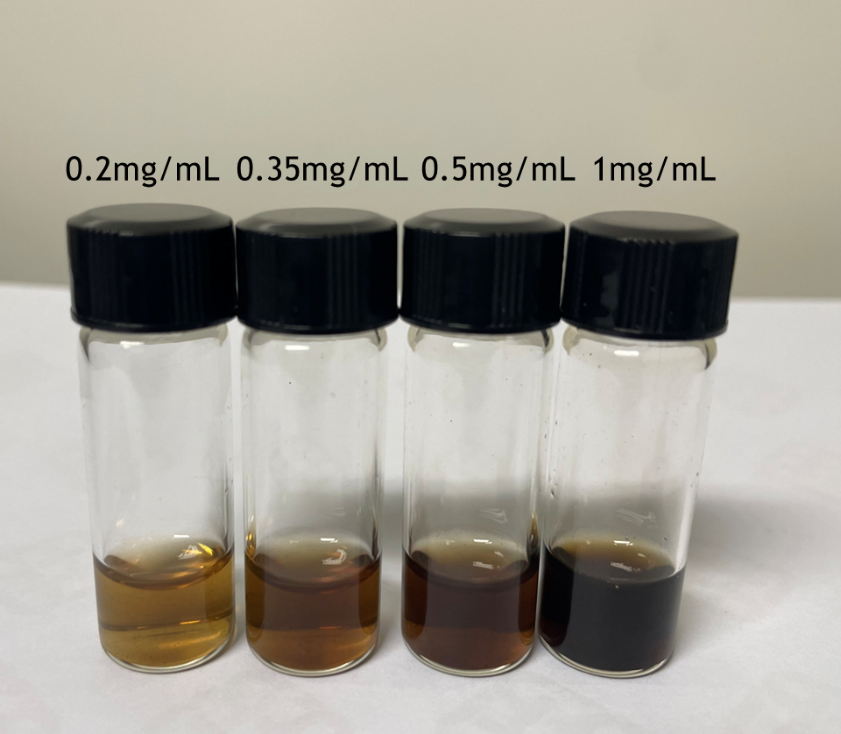


**Figure S14**. Digital picture of GO dispersions in DI water with various concentrations.


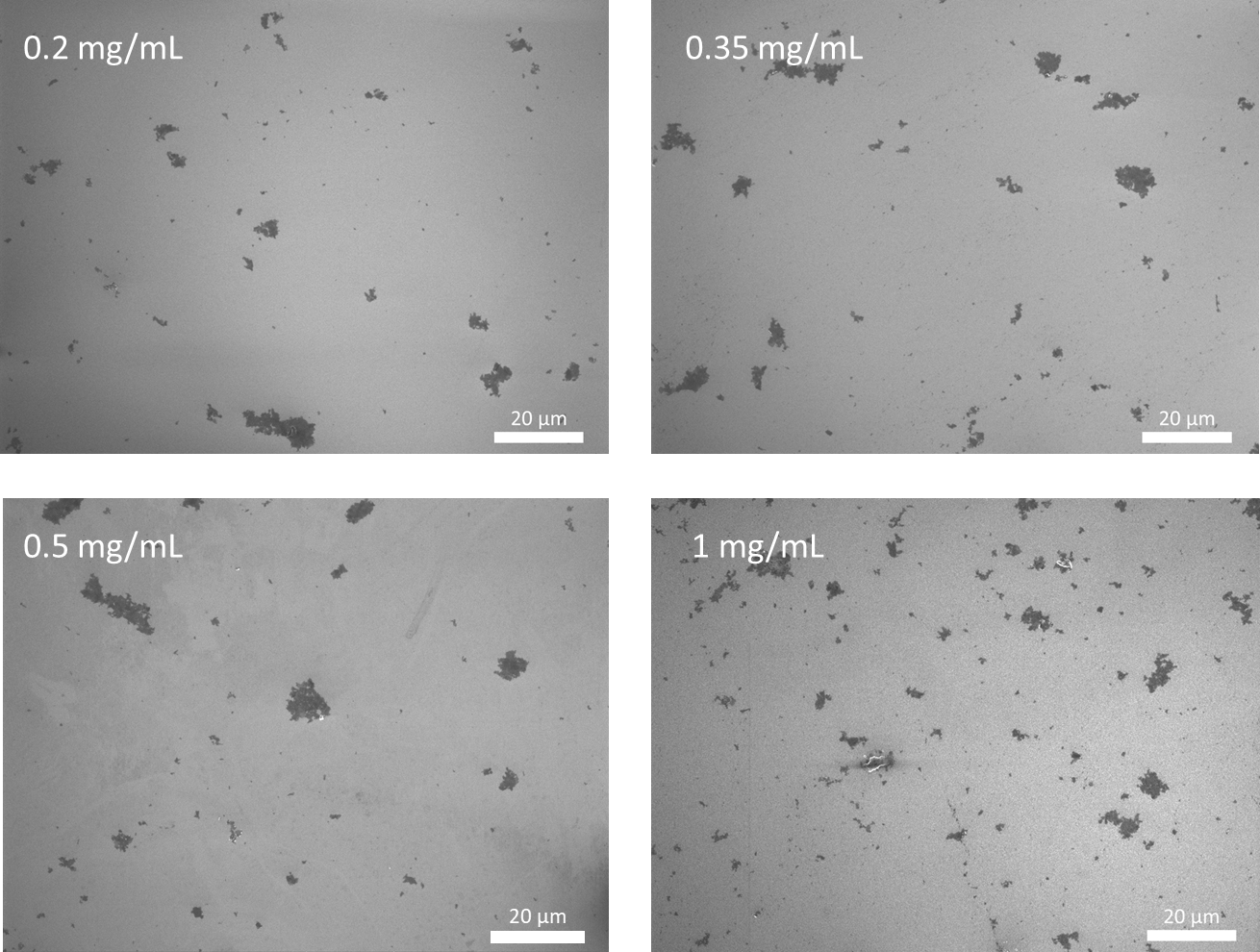
l

**Figure S15.** Top-view SEM images of spin-coated go layers on silicon substrate, prepared from GO dispersions at varying concentrations.


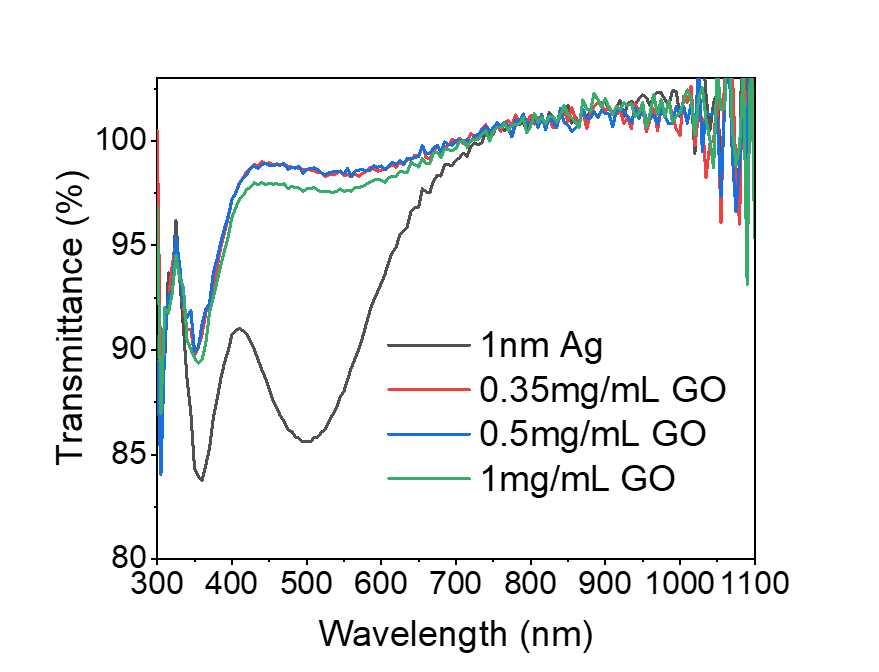


**Figure S16.** Transmittance of spin-coated GO films and thermally evaporated Ag film on ITO substrate.


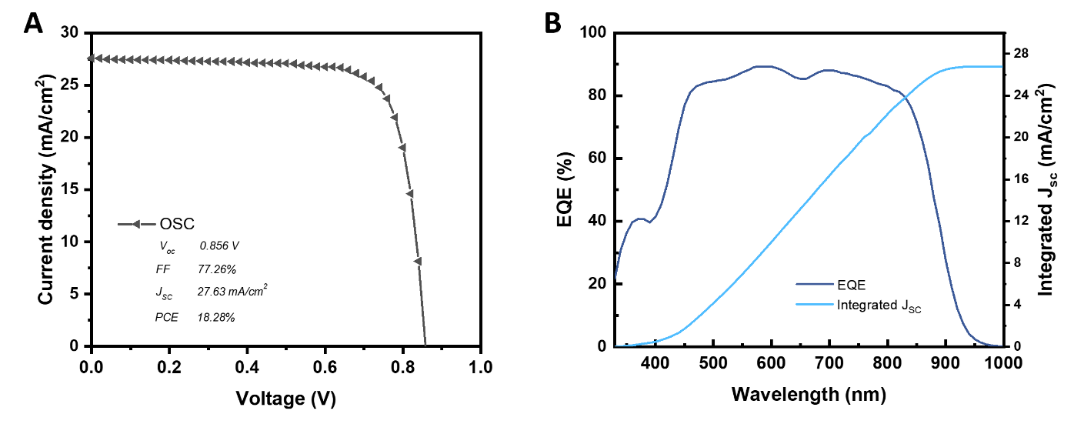
**Figure S17.** The *J-V* curve (A) and EQE spectra (B) of single-junction OPV.

**
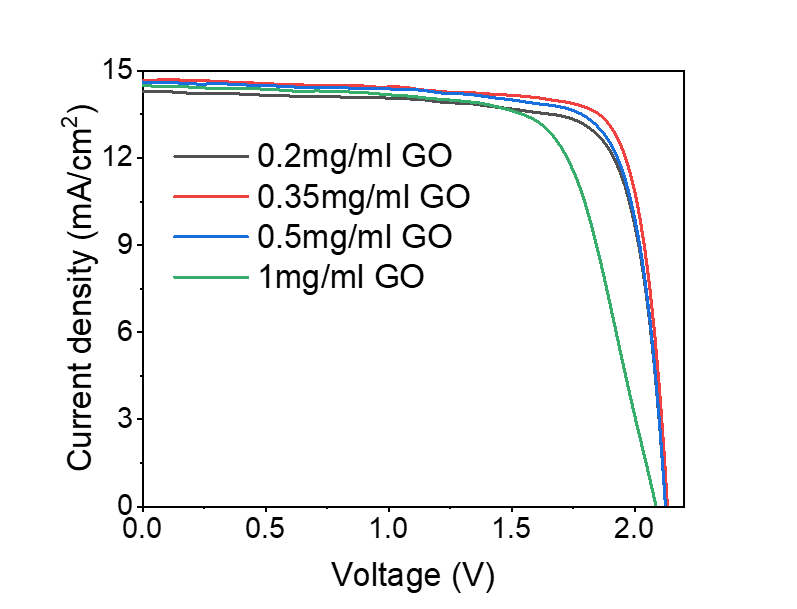
**

**Figure S18.** *J-V* curves of the PO-TSCs with different GO concentrations.


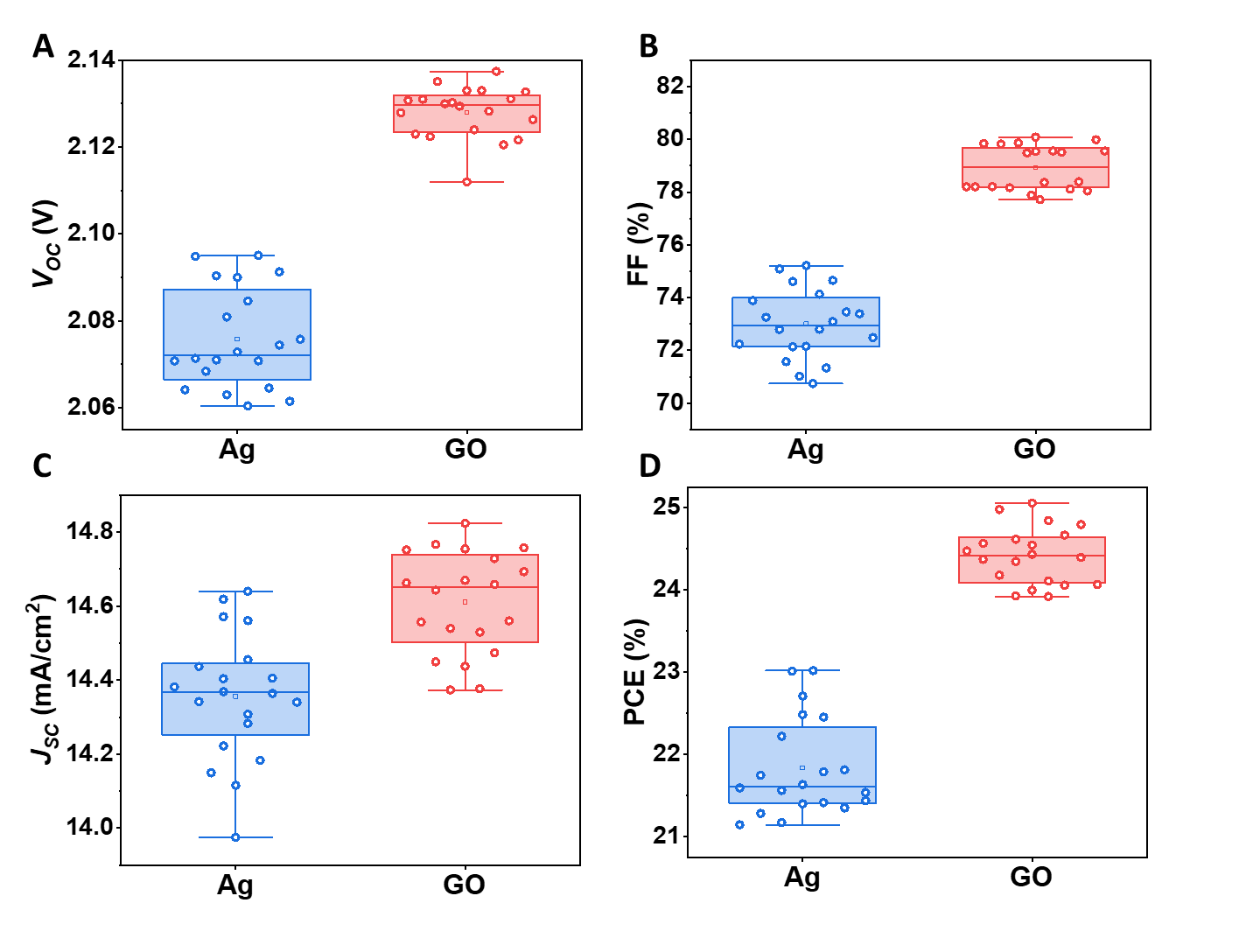


**Figure S19.** Statistics of (A) *V*_OC_, (B)FF (C) *J*_SC_ and (D) PCE for 20 POTSCs with Ag and GO as ICLs. Statistical parameters were analyzed for both Ag and GO devices (aperture area = 0.1 cm²) across 20 devices from four independent batches. The boxplots display: whiskers (data minima and maxima), box boundaries (25th and 75th percentiles), and center line (mean value).


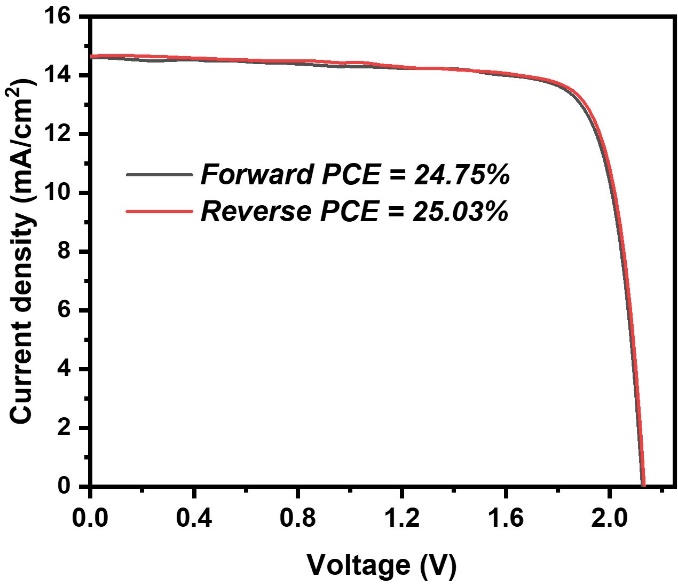


**Figure S20.** Forward and reverse scan *J–V* curves of GO-based POTSCs.

**Table S1.** *J-V* results for control WBG PSCs with different aging times.

| Control | *V_OC_* (V) | *J_SC_* (mA/cm^2^) | FF (%) | PCE (%) |
| --- | --- | --- | --- | --- |
| 0h | 1.256 | 16.50 | 80.30 | 16.64 |
| 24h | 1.250 | 16.53 | 79.90 | 16.54 |
| 48h | 1.254 | 16.48 | 79.75 | 16.48 |
| 72h | 1.232 | 16.49 | 79.82 | 16.22 |

**Table S2.** *J-V* results for WBG PSCs with different surface passivation.

| Sample | *V_OC_* (V) | *J_SC_* (mA/cm^2^) | FF (%) | PCE (%) |
| --- | --- | --- | --- | --- |
| PEABr | 1.312 | 16.72 | 82.47 | 18.11 |
| PEAI | 1.311 | 16.71 | 81.59 | 17.90 |
| OAI | 1.306 | 16.16 | 81.39 | 17.20 |
| PI | 1.352 | 16.43 | 83.87 | 18.78 |
| PipEAI | 1.355 | 16.71 | 85.61 | 19.38 |

**Table S3.** *J-V* results for target WBG PSCs with different aging times.

| Target | *V_OC_* (V) | *J_SC_* (mA/cm^2^) | FF (%) | PCE (%) |
| --- | --- | --- | --- | --- |
| 0h | 1.289 | 16.60 | 80.15 | 17.15 |
| 24h | 1.320 | 16.75 | 82.01 | 18.13 |
| 48h | 1.339 | 16.71 | 84.11 | 18.82 |
| 72h | 1.355 | 16.71 | 85.61 | 19.38 |

**Table S4.** *J-V* results for control fresh and aged WBG PSCs with different scan directions.

| Control | *V_OC_* (V) | *J_SC_* (mA/cm^2^) | FF (%) | PCE (%) |
| --- | --- | --- | --- | --- |
| 0h forward | 1.250 | 16.28 | 78.18 | 15.90 |
| 0h reverse | 1.256 | 16.50 | 80.30 | 16.64 |
| 72h forward | 1.193 | 16.35 | 78.94 | 15.39 |
| 72h reverse | 1.232 | 16.49 | 79.82 | 16.22 |

**Table S5.** *J-V* results for target fresh and aged WBG PSCs with different scan directions.

| Target | *V_OC_* (V) | *J_SC_* (mA/cm^2^) | FF (%) | PCE (%) |
| --- | --- | --- | --- | --- |
| 0h forward | 1.273 | 16.45 | 78.95 | 16.66 |
| 0h reverse | 1.289 | 16.60 | 80.15 | 17.15 |
| 72h forward | 1.352 | 16.68 | 84.77 | 19.12 |
| 72h reverse | 1.355 | 16.71 | 85.61 | 19.38 |
